# Which vaccination strategy against COVID-19?

**DOI:** 10.1093/inthealth/ihac023

**Published:** 2022-05-27

**Authors:** Alessandro De Matteis, Fethiye B Turkmen Ceylan, Enrico Urpis

**Affiliations:** University of East Anglia, School of International Development, Norwich NR4 7TJ, UK; International Society of Global Health, Edinburgh EH3 8HE, UK; University of East Anglia, School of International Development, Norwich NR4 7TJ, UK; Asociación Latina para el Análisis de los Sistemas de Salud, Barcelona 08006, Spain

**Keywords:** COVID-19, dose sparing, vaccination

## Abstract

**Background:**

Bottlenecks in the production and supply pipeline of vaccines against coronavirus disease 2019 have led some countries to consider the option of dose-sparing strategies (e.g., increasing the number of people who receive some vaccine by halving the dose or increasing the interval between doses). In this study we assess the contribution of vaccination strategies to reducing the mortality induced by severe acute respiratory syndrome coronavirus 2.

**Methods:**

This study focuses on the evolution of the pandemic and related vaccination efforts in five countries that have adopted different vaccination strategies or have experienced a bottleneck in their vaccine supply. The analysis is conducted using an autoregressive time-series approach through a system of simultaneous equations.

**Results:**

The outcome of the early months of the vaccination campaign in containing the number of deaths induced by the epidemic varies across our sample. Overall, our results highlight the effective role played by the vaccine in containing the death toll induced by the epidemic. We could not find evidence of reduced effectiveness of the second dose in the presence of an extended inter-dose interval. The effectiveness of the vaccination campaign results appears to be strongly affected by the stability of vaccine supply.

**Conclusions:**

The vaccine is effective in containing the deaths caused by the virus, particularly when multiple doses have been administered. The stability of the vaccine pipeline plays a critical role in determining the effectiveness of the vaccination campaign.

## Introduction

Early on in the vaccination effort, parallel to the topics of vaccine development and of selection of the most suitable option among the available vaccine alternatives,^1,2^ the focus of debate was on how to structure the vaccination strategy, in particular regarding (a) prioritizing some categories of the population over others (e.g., the retired vs those of working age) and (b) the administration of the second dose.

On the first topic, as well as the presentation and updating of various governments’ vaccination strategies with details about calendars and priority groups^3,4^ and technical studies illustrating the trials of the different vaccines,^5^ in the very early stages of the vaccination campaign some researchers sought to develop mathematical models in an attempt to simulate likely scenarios. Many of these were based on strict assumptions about model parametrization and often ended up with conflicting results. Moore et al. and Gog et al. provide an example of the extreme sensibility that affects mathematical simulations. As they both define their models with a set of parameters, they come up with opposite results.^6,7^ Hence the urge to rely on empirical studies to overcome such drawbacks. Other studies estimated the number of years of life lost together with empirical mortality rates, remaining life expectancy and person-years saved per vaccination.^8^ Once real-world data started to surface, the analysis turned to the experiences of the countries leading the vaccination campaign, highlighting the vaccine's effectiveness in preventing symptomatic and asymptomatic severe acute respiratory syndrome coronavirus 2 (SARS-CoV-2) infections and related hospitalizations, severe disease and death across diverse populations in a non-controlled setting.^9-11^

On the second topic, vaccine manufacturers provided their own recommendations about the timing of the second dose; the three vaccines approved earliest recommended: 21 d apart (Pfizer-BioNTech), 28 d (Moderna) and 4-12 wk apart (Oxford-AstraZeneca). However, bottlenecks in the production and supply pipeline resulted in the consideration of dose-sparing strategies. The initial article^12^ urging single dose interim use to extend vaccination to as many people as possible until vaccine availability improved was followed by several others, igniting heated debate. Some countries, notably the UK and Canada, adopted a policy prioritizing administering a first dose over giving second doses (in the UK, the delivery plan was adjusted on 30 December 2020). Other countries extended the inter-dose interval or considered giving two half-doses, while others still, such as France and Italy, decided not to delay the second dose—more recently, an increasing number of countries that initially followed the protocol proposed by the vaccine producers have extended the inter-dose interval—but faced severe breaks in vaccine supply with consequent delays to their campaigns.^13,14^ Finally some countries, notably Israel and Germany, preferred to adhere to the vaccine producers’ recommendations, making sure to avoid breaks in the pipeline. Israel, thanks also to its limited population size, was able to secure a stable pipeline by agreeing to pay relatively high prices for the vaccine and providing the manufacturers with free access to a large dataset of personal data, making it possible to use data on Israeli citizens for the final stages of field testing and rolling out of the vaccination campaign. Germany managed to secure the number of doses required for its population of 83 million. In fact, while championing the European Union’s joint purchasing of coronavirus vaccines in the second half of 2020, Germany managed to secure far more than its pro-rata allocation by purchasing leftover doses and, on top of that, also made additional agreements with vaccine producers, securing extra doses.^15^

Multiple studies have suggested that dose-sparing strategies would reduce the burden of disease in the epidemic^16-18^ and have reported that a single dose of vaccines offers a high level of protection against mortality, supporting the option of prioritizing at-risk groups for the first dose in the context of high disease incidence and constraints to vaccine supply or delivery.^19^

Conflicting views have been expressed about the possibility that dose-sparing could increase the risk related to vaccine-escape variants. While one side argues that dose-sparing will cause the more rapid emergence and spread of vaccine-resistant genetic variants,^20^ other views suggest that it could reduce the spread of vaccine-resistant variants rather than increase it.^21^

In this study we assess the contribution of vaccination strategies to reducing SARS-CoV-2–induced mortality. Our empirical approach is expected to overcome the contradictory results deriving from theoretical analysis and to provide a solid base that can have important implications in fighting against coronavirus disease 2019 (COVID-19) globally and in particular for developing countries where the supply of vaccines has not been satisfactory so far.

## Methods

### Data

We use data from Johns Hopkins University's COVID-19 data repository^22^ on the number of deaths due to the epidemic, the number of detected cases and the number of vaccine doses administered in a sample of five countries: France, Germany, Israel, Italy and the UK. All data used in this study have daily frequency in absolute terms and have been logarithmically transformed. Hence, even short series of high-frequency data can help to highlight functional relationships, which, otherwise, would be hidden by the delayed evolution of the infection from its early stage into a full-blown case. The dataset provides the number of first and second doses administered; eventual cases of reinfection are not identified, however, the short period covered by this study makes it unlikely that such cases may have occurred within the timeframe considered.^23^

The study covers the period from 1 December 2020 to 20 April 2021, when the European Medicines Agency authorized the use of the first mono-dose vaccine.

### Analytical approach

Vaccination affects mortality in two ways: it contributes to containing the spread of the disease, with a consequent reduction of the related death toll, and it can reduce the death toll from the virus by decreasing its infectiousness.^24^ In other words the number of deaths is expected to decrease with vaccination for two reasons: first, the vaccine will reduce the number of new cases, and second, new cases will involve a lower risk of death. A model is required to address both of the ways in which the vaccine can effectively reduce the risk of death. For this purpose we apply a system of simultaneous equations in which the number of new deaths due to COVID-19 is our dependent variable, and the number of new cases along with the number of vaccines administered daily are our explanatory variables.

An autoregressive approach is required to take account of the daily evolution of the events analyzed in this study. However, this raises a methodological issue. Each model related to a country in the panel is in itself a classical regression where parameters are estimated consistently and efficiently, one equation at a time, using ordinary least squares. The assumption of independence of the error terms in each equation implies that no common factors influence all the countries. In our case it would be naïve to assume this, because the COVID-19 pandemic is global and all countries covered in the study are embedded in the same structure, and therefore may be intrinsically affected by common factors not addressed in the model. Therefore, a seemingly unrelated regression (SUR) model is appropriate.^25^ SUR is a type of generalized linear regression model that assumes that the error terms of the equations are correlated, implying that there are common observable factors for all equations. Hence, SUR provides consistent and efficient parameter estimates. We use the Breusch–Pagan test to ascertain this.

The advantage of using SUR is twofold: it allows the estimation of unbalanced time series equations simultaneously, and it delivers more efficient parameter estimates (i.e., achieves smallest variance) when the data are limited or heterogeneity in the data cannot be properly addressed due to unobserved factors. In our case, all the countries in our sample experienced constraints to their access to the vaccines due to supply management. Therefore we should take into account some common factors that can lead to heteroscedasticity in the data, and are required to use a generalized least squares estimator that provides robust standard errors, such as in the SUR model.

The elasticity in our case implies the responsiveness of the number of new deaths from new cases of COVID-19 to the first or second dose of the vaccine. To capture elasticity, the dependent and independent variables are logarithmically transformed, hence a log model is estimated. The functional form of the equation is presented below:

Models about the impact of the first dose:

(1.1) \begin{eqnarray*} \left[ \begin{array}{@{}l@{}} newdeath{s_{1,t}}\\ \vdots \\ newdeath{s_{5,t}} \end{array} \right] = \left[ \begin{array}{@{}l@{}} {c_1}\\ \vdots \\ {c_5} \end{array} \right] + \alpha \left[ \begin{array}{@{}l@{}} newcase{s_{1,t}}\\ \vdots \\ newcase{s_{5,t}} \end{array} \right] \\ +\, \beta \left[ \begin{array}{@{}l@{}} firstdos{e_{1,t}}\\ \vdots \\ firstdos{e_{5,t}} \end{array} \right] + \left[ \begin{array}{@{}l@{}} {u_1}\\ \vdots \\ {u_5} \end{array} \right] \end{eqnarray*}

Models about the impact of both doses:

(1.2) \begin{eqnarray*} \left[ \begin{array}{@{}l@{}} newdeath{s_{1,t}}\\ \vdots \\ newdeath{s_{5,t}} \end{array} \right] = \left[ \begin{array}{@{}l@{}} {c_1}\\ \vdots \\ {c_5} \end{array} \right] + \alpha \left[ \begin{array}{@{}l@{}} newcase{s_{1,t}}\\ \vdots \\ newcase{s_{5,t}} \end{array} \right] \\ +\, \beta \left[ \begin{array}{@{}l@{}} bothdoses{_{1,t}}\\ \vdots \\ bothdoses{_{5,t}} \end{array} \right] + \left[ \begin{array}{@{}l@{}} {u_1}\\ \vdots \\ {u_5} \end{array} \right] \end{eqnarray*}

where *c* is the constant in each equation, *α* measures the responsiveness of the number of new deaths to the number of new cases and *β* measures the responsiveness of the number of new deaths to first and second doses of the vaccine. Lastly, *u* is the residual of the equations that will be tested against normality, autocorrelation and heteroscedasticity.

To take account of the possible risk of endogeneity among the variables considered in the models proposed above, sensitivity analysis has been conducted through a vector error correction (VEC) approach (resources about VEC can be found at https://www.stata.com/manuals/tsvecintro.pdf).

## Results

**Figure 1.**
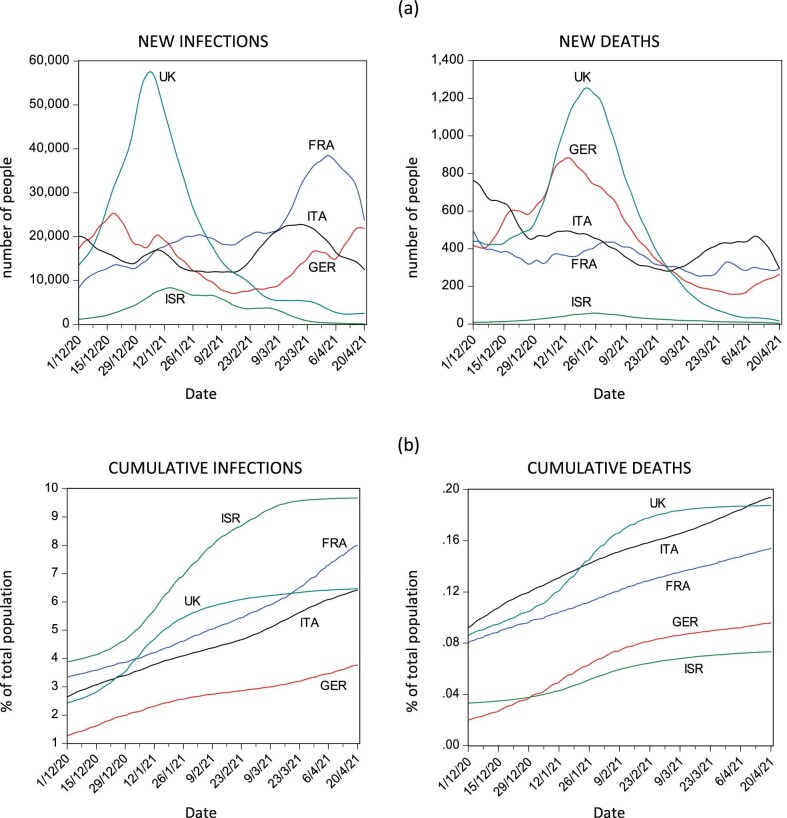
(a) New infections and deaths due to COVID-19 and (b) cumulative caseloads of infections and deaths due to COVID-19 as percentage of total population in a sample of countries from 1 December 2020 to 20 April 2021.

Figure 1 shows the patterns of contagion and of COVID-19–related death caseload in the five countries considered. Beyond the general similarities across all countries, it is possible to identify two groups in terms of evolution of cumulative infections: while France, Germany and Italy show an approximately linear trend, a visible hump can be spotted in the cases of the UK and Israel, resembling an S-shaped evolution. Very briefly, the situation in the latter group can be explained in light of the facts that (a) the initial high prevalence of the disease in Israel contributed to accelerating the spread of the virus among its relatively small population, and (b) the spread of the British variant of the virus accelerated the increase in cases in the UK during the winter period. This is clearly reflected in the peak of new infections recorded in the UK. In both cases the drastic halt to the epidemic's rapidly increasing rate of progression can be explained by the aggressive mass-vaccination campaigns implemented in the two countries.

**Figure 2.**
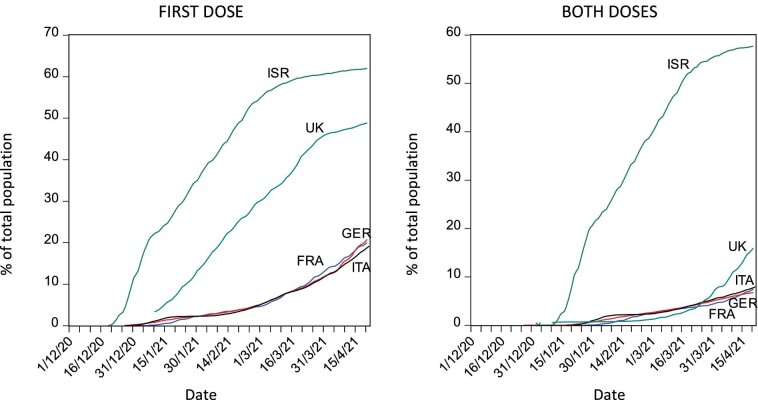
First-dose and both-doses vaccination coverage as percentage of total population in a sample of countries from 1 December 2020 to 20 April 2021.

Figure 1 also shows the evolution of the death toll caused by the epidemic in the five countries. In France, Germany and Italy we see again the upward trend already encountered in the number of infected cases. The cumulative number of COVID-19–related deaths in the UK closely mirrors the S-shaped evolution of the infection caseload. The evolution of COVID-19–related deaths in Israel is also S-shaped, but smoother than the evolution of the infection caseload. It is interesting to consider that while Israel regularly records the highest caseload of infected cases as a share of the overall population among the countries considered, almost throughout the whole of the period considered here Israel accounts for the lowest number of deaths relative to its population. In other words, the rapid spread of the disease among the Israeli population led to a much-less-than-proportional increase in COVID-19–related deaths.

Interestingly, the evolution of new COVID-19–related deaths reveals some resemblance among the UK, Germany and, in relative terms, also Israel. On the contrary, the evolution of new death cases occurred in Italy shows a decreasing trend, while it remains rather stable in France.

The different age structure of the populations is expected to play a role in this regard, all other things remaining equal. It is argued that COVID-19–related mortality increases significantly with age,^26^ and therefore, despite its highest prevalence of the disease, Israel, with its younger population, faced a smaller death toll than the other countries in our sample. Among all the countries in our sample, Israel has the lowest median age (30 y), whereas this variable reaches its maximum value in Italy (47 y), followed in decreasing order by Germany (45 y), France (42 y) and the UK (41 y).

Besides the age structure of the population, other factors are expected to play a role in influencing COVID-19–related mortality. In particular, our focus here is on the vaccination strategy. Israel and the UK were front runners with their mass vaccination campaigns, with the only major difference being that Israel maintained the manufacturer-recommended inter-dose interval, while the UK adopted a dose-sparing strategy by imposing a longer inter-dose interval.

As shown in Figure 2, the UK's dose-sparing strategy allowed it to achieve a remarkable first-dose vaccination performance, mirroring the results achieved by Israel over a much smaller population and leaving the modest performance achieved by the other three countries in our sample behind. As expected, the drawback of the dose-sparing strategy adopted is evident when considering the results achieved regarding the administration of the second dose, with the UK performing quite similarly to France, Germany and Italy and far behind Israel. By 20 April, a remarkable 58% of the Israeli population had received the second dose compared with 16% of the UK population and only 7–8% in Italy, Germany and France, in decreasing order.

At this stage we have run the equations presented in (1.1) and (1.2) to analyze the contribution of first and second doses of vaccine to the occurrence of new deaths. Table 1 presents the estimation results from the two models. The models for the UK, Israel and Germany have an acceptable degree of significance, as indicated by the values of the adjusted R^2^, while those for France and Italy are less reliable. For both systems of equations both the Breusch–Pagan tests and the correlation matrix of residuals—presented in Table A.1 in the Appendix—confirm the efficiency of the SUR estimation.

**Table 1.** Seemingly unrelated regression analysis on the effectiveness of vaccine doses over new deaths

Dependent variable: number of new deaths											
First model						Second model					
Indepent variables	France	Germany	Israel	Italy	UK	Indepent variables	France	Germany	Israel	Italy	UK
First dose	0.02	−0.59 ***	−0.67 ***	−0.03	0.68 ***	Both doses	−0.06 **	−0.81 ***	−1.10 ***	0.05	−0.89 ***
New cases	−0.09	0.48	0.39 ***	0.18 **	2.00 ***	New cases	−0.06	0.46 ***	0.27 ***	0.06	0.63 ***
Constant	6.20 ***	10.00 ***	10.00 ***	4.60 ***	−24.00 ***	Constant	7.20 ***	13.00 ***	17.00 ***	4.60 ***	12.00 ***
Adj. R^2^	0.035	0.464	0.801	−0.007	0.837	Adj. R^2^	0.105	0.457	0.846	0.019	0.862
Breusch–Pagan Pr			0.00***			Breusch–Pagan Pr			0.00***		

Significance: *** p<0.01, ** p<0.05

As expected, our results indicate that the occurrence of new cases leads to new deaths. The relationship between the occurrence of new cases and new deaths is consistently stronger in the UK than in Germany, followed by Israel and Italy. Here it is evident the impact of the B.1.1.7 (Delta) variant which was spreading quickly in the UK and Israel during the period under examination. Although no consensus has yet been reached on whether the Delta variant is associated with substantially higher mortality, it is worth considering that a set of studies of this variant have estimated an increased HR for the risk of death of 61–67%.^27-29^ Considering the high contagion and lethality attributed to the Delta variant, it is plausible to ascribe to this variant the high value found for the coefficient related to the impact of new cases over new deaths in the first-dose UK model. Any increase in the number of new cases in the UK is associated with a rise in COVID-19–related deaths that is approximately four- and 10-fold that of Germany and Italy, respectively.

Along the same lines, the positive sign of the coefficient related to the effectiveness of the first dose in contrasting the lethality of the virus in the UK reveals that the spread of the Delta variant was faster than the speed at which vaccination was conducted in the UK. In fact the bulk of first-dose vaccinations in the UK took place when the Delta variant was already dominant and spreading faster than how the vaccination campaign was being rolled out.

With the exception of Italy and partially of France, both the first and second dose have a statistically significant effect in reducing the number of deaths, which appears to be particularly effective in Israel and the UK, followed by Germany. Also, as expected, the impact of two doses on COVID-19–induced mortality is greater than the effect generated by the first-dose vaccine. This plausible finding is supported by the consideration that in all the countries in our sample the contribution of new cases to the mortality caused by COVID-19 decreases after the second dose of the vaccine. This consideration also applies to the Delta variant.^30,31^

As highlighted earlier, the equations for France and Italy provide only a weak explanation of the determinants of new COVID-19–related deaths. While for France only the second dose is found to play a significant role in curbing the COVID-19–related death toll, in the case of Italy the vaccines are not found to have a statistically significant impact on the number of new deaths. This finding suggests the lack of a consistent vaccination strategy in these two countries, a consideration that finds its major support in the uncertainties due to the on-off supply of vaccines during the period under consideration. Having said that, the limited fit of models for data from France and Italy raises questions about the modest impact—or lack of impact—that in both countries new cases and vaccination are found to have on new deaths. The distribution of the model residuals and error structure seems to hint at the possibility that some important explanatory variables have not been considered in the analysis. Deaths due to COVID-19 are known to be associated with other factors, such as age of the population, baseline death rate and related morbidity and general conditions of vulnerability. Likewise, non-pharmacological strategies aimed at containing the spread of the epidemics—for example, virus testing, social distancing and measures of lockdown, among others—are expected to effectively break the chain of transmission and contain the spread of the virus.

Both sets of variables are expected to affect the effectiveness of vaccination over the progression of the epidemic and over its death toll. Nevertheless, they can also play a role of confounding factors, particularly when comparing the dynamics of the epidemics and a diverse set of containment strategies within a group of slightly different populations. As shown in Table A.2 in the Appendix, data about life expectancy and age groups indicates that France and, in particular Italy, are among the countries in our sample with the largest populations of older people. However, a few morbidity indicators that are relevant to our analysis do not reveal a higher degree of vulnerability about the French and Italian people. On the contrary, Italy performs poorly among the countries in our sample in terms of health expenditure; nevertheless under this perspective the performance of Israel is worse.^32-34^ Likewise, data about the implementation of some non-pharmacological measures, shown in Table A.3, provide contradictory information. While Italy has regularly put in place stricter measures than France in terms of lockdowns, school attendance and public transport, the strictest control measures imposed within our sample occurred in the UK and Germany.^35^ Hence, while on one hand it is reasonable to expect that both the structural features of the populations considered in our sample as well as the different non-pharmacological measures put in place in the various countries have contributed to affect the spread of the epidemic and consequently its death toll, on the other hand the different ways in which all such factors operate end up playing a confounding role on the focus of our study, that is, the relevance of the vaccination strategy.

At this stage, for the purposes of sensitivity analysis, the estimations conducted so far have been replicated through a VEC approach, which allows to take account of the possible risk of endogeneity.

For all countries in our sample a long-run relationship between the number of new cases, new deaths and the number of vaccine doses administered is found, and the coefficients in Table 2 provide general support to the results of the SUR analysis presented earlier on. The effective role played by the vaccine in containing the death toll induced by the epidemic is confirmed for all countries in our sample. Contrary to results in Table 1, new results highlight that vaccination has also played a significant contribution against COVID-19–related mortality in France and Italy. Having said that, the insignificant coefficient related to the first dose administered in Italy keeps indicating the difficulties due to the instability of the vaccine pipeline during the early stages of the vaccination campaign.

**Table 2.** VEC analysis on the effectiveness of vaccine doses over new deaths

Dependent variable: number of new deaths											
First model						Second model					
Indepent Variables	France	Germany	Israel	Italy	UK	Indepent Variables	France	Germany	Israel	Italy	UK
First dose_t-1_	−1.05***	−0.45	−28.47***	−0.03	−2.68*	Both doses_t-1_	−0.17***	−0.82**	1.34***	−0.14**	−2.62*
New cases_t-1_	3.20***	1.24*	−0.29	−0.12	−4.68***	New cases_t-1_	0.27***	6.21**	−0.29***	−0.43***	−10.96***

Significance: *** p<0.01, ** p<0.05, * p<0.1

Note: As the process of cointegration is expected to follow different patterns in each of the countries considered, the lag length was selected separately in each model. The available information criteria led to adopting optimum lag lengths of 4–9 d.

Interestingly, the new results related to Israel highlight a sharp contrast between a very effective first dose and an apparently ineffective second dose of vaccine. In this case the longer lags applied by the VEC modeling approach than by the SUR one have captured the effect of the fast-spreading Delta variant: despite the robust vaccination strategy adopted in Israel, administration of the second dose could not keep pace with the spread of the new variant.^36^ Likewise, the longer lag length adopted by the VEC estimation highlights that the vaccine's protection capacity boosts over time; in fact, the estimated negative effects of new cases on new deaths in the models about the UK, Israel and Italy indicate that the vaccine is effective in containing the case fatality rate.

## Discussion and conclusions

The analysis conducted in this study has empirically addressed the question of which COVID-19 vaccination strategy has proven to be most effective.

Israel's strategy to combine massive vaccination coverage with maintaining the recommended inter-dose interval has proven to be the most successful. While its efforts are supported by its limited population size, Germany, with its much larger population, has achieved similar results. As for the UK, our analysis could not find evidence of reduced effectiveness of the second dose in the presence of an extended inter-dose interval.

The results of France and Italy's early stages of vaccination campaigns highlight the critical contribution of the stability of the vaccine pipeline to the efficacy of the vaccination campaign.

Although this study is focused on the experience of a few countries, its findings are expected to have wider application. In particular, they may support the design of vaccination strategies for those countries where proper vaccination campaigns are still to be launched or are at an early stage.

Since our analysis has highlighted a confounding effect caused by the non-pharmacological measures adopted in different countries to contain the spread of the virus, we recommend further research on the combined use of pharmaceutical and non-pharmaceutical measures in curbing the death toll induced by the epidemic.

Finally, as a limitation of this study, it is necessary to consider that the analysis is based on aggregated secondary data and hence a word of caution is appropriate in result interpretation.

## Data Availability

Data sources are provided.

## Appendix

**Table A.1.** Correlation matrix of residuals

*Only first dose*	*France*	*Germany*	*New deaths in: Israel*	*Italy*	*UK*
*New deaths in:*					
*France*	1.000				
*Germany*	0.715	1.000			
*Israel*	−0.135	0.021	1.000		
*Italy*	0.520	0.299	−0.089	1.000	
*UK*	0.054	0.078	0.134	−0.169	1.000
			*New deaths in:*		
*Both doses*	*France*	*Germany*	*Israel*	*Italy*	*UK*
					
*New deaths in:*					
*France*	1.000				
*Germany*	0.716	1.000			
*Israel*	−0.278	−0.160	1.000		
*Italy*	0.523	0.271	−0.052	1.000	
*UK*	0.235	0.086	−0.052	0.327	1.000

**Table A.2.** Duration of some non-pharmacological measures (% of period considered in this study)

	*France*	*Germany*	*Israel*	*Italy*	*UK*
*School attendance*					
Not in place	0	0	0	0	0
Recommend closing schools	44	11	0	0	10
Require closing only some levels or categories of schools	56	0	100	62	26
Require closing all levels of schools	0	89	0	38	64
*Workplaces*					
Not in place	0	0	0	0	0
Require closing (or work from home) for some sectors or categories of workers	75	43	66	47	14
Require closing (or work from home) for all-but-essential workplaces	25	57	34	53	86
*Public events*					
Not in place	0	0	0	0	0
Require cancelling	100	100	100	100	100
*Gatherings*					
Not in place	0	0	0	0	0
Restrictions on gatherings between 11–100 people	0	0	55	0	1
Restrictions on gatherings of 10 people or less	100	100	45	100	99
*Public transportation*					
Not in place	100	0	11	0	0
Recommend closing or significantly reduce volume of transport available	0	100	89	100	100
*Stay-at-home requirements*					
Not in place	0	0	46	0	4
Recommend not leaving house	0	11	0	0	10
Require not leaving house with exceptions for 'essential' trips	100	89	54	100	86
*Internal movement*					
Not in place	64	0	46	0	0
Recommend not to travel between regions/cities	0	44	0	0	11
Internal movement restrictions in place	36	56	54	100	89
*International travel controls*					
Not in place	0	0	0	0	0
Quarantine arrivals from some or all regions	0	0	0	0	18
Ban arrivals from some regions	100	100	16	100	82
Ban on all regions or total border closure	0	0	84	0	0

Source: Author's analysis of data from Hale et al., 2021

**Table A.3.** Main health indicators*

	*France*	*Germany*	*Israel*	*Italy*	*UK*
*Demography*					
Life Expectancy (yrs)^§^	82.7	80.9	82.8	83.3	81.3
Population over 60 years (%)′	26.1	28.1	16.2	29.0	23.9
Population over 80 years (%)′	6.2	6.6	3.1	7.2	5.0
*Morbidity*					
Share of adults with diabetes (%)^§^	5.4	5.3	7.4	5.1	5.2
Prevalence of anemia, in older people, Yrs 95+ (%)′	11.6	17.2	21.9	15.7	19.2
Asthma hospital admissions, Yrs 15+ (age-sex standardised rate per 100 000 population)′′	30.1	28.7	49.9	7.6	71.0
*Health indicators*					
Health expenditure per capita (US${\$}$ PPP)^§^	5250	6098	3207	3624	4619
Health expenditure as % of GDP^§^	11.3	11.4	7.5	8.7	10.0

Note:*2018 or nearest year

Source: Author's analysis of data from: ^§^WB, ‘ WHO, “ OECD

## References

[1] Abdelwahab SF , Issa UH, Ashour HM. A novel vaccine selection decision-making model (VSDMM) for COVID-19. Vaccines. 2021;9:718. 34358134 10.3390/vaccines9070718 PMC8310225

[2] Forestal LF , Pi S-M. A hybrid approach based on ELECTRE III-genetic algorithm and TOPSIS method for selection of optimal COVID-19 vaccines. J Multi-Criteria Decision Analysis. 2021;29:80–91. 10.1002/mcda.1772 PMC8646624 40477443

[3] European Centre for Disease Prevention and Control . Overview of the implementation of COVID-19 vaccination strategies and deployment plans in the EU/EEA –14 June 2021. Stockholm, Sweden: ECDC; 2021.

[4] Bingham K . The UK Government's Vaccine Taskforce: strategy for protecting the UK and the world. Lancet North Am Ed. 2021;397(10268):68–70. 10.1016/S0140-6736(20)32175-9 PMC7833709 33125932

[5] Voysey M , Clemens SAC, Madhi SA et al. Safety and efficacy of the ChAdOx1 nCoV-19 vaccine (AZD1222) against SARS-CoV-2: an interim analysis of four randomised controlled trials in Brazil, South Africa, and the UK. Lancet North Am Ed. 2021;397(10269):99–111. 10.1016/S0140-6736(20)32661-1 PMC7723445 33306989

[6] Moore S , Hill EM, Dyson L, Tildesley MJ, Keeling MJ. Modelling optimal vaccination strategy for SARS-CoV-2 in the UK. PLoS Comput Biol. 2021;17(5):e1008849. 33956791 10.1371/journal.pcbi.1008849 PMC8101958

[7] Gog JR , Hill EM, Danon L, Thompson R. Vaccine escape in a heterogeneous population: insights for SARS-CoV-2 from a simple model. R Soc Open Sci. 2021;8(7):210530. 34277027 10.1098/rsos.210530 PMC8278051

[8] Goldstein JR , Cassidy T, Wachter KW. Vaccinating the oldest against COVID-19 saves both the most lives and most years of life. Proc Natl Acad Sci. 2021;118(11):e2026322118. 33632802 10.1073/pnas.2026322118 PMC7980436

[9] Rossman H , Shilo S, Meir T, Gorfine M, Shalit U, Segal E. COVID-19 dynamics after a national immunization program in Israel. Nat Med. 2021;27(6):1055–61. 33875890 10.1038/s41591-021-01337-2

[10] Haas EJ , Angulo FJ, McLaughlin JM et al. Impact and effectiveness of mRNA BNT162b2 vaccine against SARS-CoV-2 infections and COVID-19 cases, hospitalizations, and deaths following a nationwide vaccination campaign in Israel: an observational study using national surveillance data. Lancet North Am Ed. 2021;397:1819–28. 10.1016/S0140-6736(21)00947-8 PMC8099315 33964222

[11] Dagan N , Barda N, Kepten E et al. BNT162b2 mRNA Covid-19 vaccine in a nationwide mass vaccination setting. N Engl J Med. 2021;384:1412–23. 33626250 10.1056/NEJMoa2101765 PMC7944975

[12] Plotkin SA , Halsey N. Accelerate coronavirus disease 2019 (COVID-19) vaccine rollout by delaying the second dose of mRNA vaccines. Clin Infect Dis. 2021;73(7):1320–1. 33502467 10.1093/cid/ciab068 PMC7929065

[13] Jewkes S . Italy to take legal action on COVID vaccine delays to get doses. Reuters. 2021.

[14] Paccalin C . Facing shortages, French medical centres forced to delay COVID-19 vaccinations. France24. 2021.

[15] Deutsch J , Furlong A, Von Der Burchard H, Martuscelli C. Thanks to deep pockets, Germany snaps up extra coronavirus jabs. Politico. 2021.

[16] Tuite AR , Zhu L, Fisman DN, Salomon AJ. Alternative allocation strategies to increase benefits from constrained COVID-19 vaccine supply. Ann Intern Med. 2021;174(4):570–2. 33395334 10.7326/M20-8137 PMC7808325

[17] Barnabas RV , Wald A. A public health COVID-19 vaccination strategy to maximize the health gains for every single vaccine dose. Ann Intern Med. 2021;174(4):552–3. 33395339 10.7326/M20-8060 PMC7808326

[18] Paltiel AD , Zheng A, Shwartz JL. Speed versus efficacy: quantifying potential tradeoffs in COVID-19 vaccine deployment. Ann Intern Med. 2021;174(4):568–70. 33395345 10.7326/M20-7866 PMC7787166

[19] Lopez Bernal J , Andrews N, Gower C et al. Effectiveness of BNT162b2 mRNA vaccine and ChAdOx1 adenovirus vector vaccine on mortality following COVID-19. MedRxiv. 2021.

[20] Bieniasz P . The case against delaying SAS-COV-2 mRNA vaccine boosting doses. Clin Infect Dis. 2021;73(7):1321–3; Saad-Roy CM, Morris SE, Metcalf CJE, et al. (2021) Epidemiological and evolutionary considerations of SARS-CoV-2 vaccine dosing regimes, *Science*. 2021;372(6540):363–70. 33688062 10.1126/science.abg8663 PMC8128287

[21] Cobey S , Larremore DB, Grad YH et al. Concerns about SARS-CoV-2 evolution should not hold back efforts to expand vaccination. Nature Review Immunology. 2021;21:330–5. 10.1038/s41577-021-00544-9 PMC8014893 33795856

[22] John Hopkins University . Coronavirus Resource Center. https://coronavirus.jhu.edu/about/how-to-use-our-data [accessed May 1, 2021].

[23] Abu-Raddad LJ , Chemaitelly H, Bertollini R. Severity of SARS-CoV-2 reinfections as compared with primary infections. N Engl J Med. 2021;385:2487–9. 34818474 10.1056/NEJMc2108120 PMC8631440

[24] Regev-Yochay G , Sharon A, Bergwerk M et al. Decreased infectivity following BNT162b2 vaccination: a prospective cohort study in Israel. The Lancet Regional Health-Europe. 2021;7:100150. 34250518 10.1016/j.lanepe.2021.100150 PMC8261633

[25] Zellner A . An efficient method of estimating seemingly unrelated regressions and tests for aggregation bias. J Am Statist Assoc. 1962;57(298):348–68.

[26] Bonanad C , García-Blas S, Tarazona-Santabalbina F et al. The effect of age on mortality in patients with COVID-19: a meta-analysis with 611,583 subjects. J Am Med Dir Assoc. 2020;21(7):915–8. 32674819 10.1016/j.jamda.2020.05.045 PMC7247470

[27] Davies NG , Jarvis CI, Edmunds WJ et al. Increased mortality in community-tested cases of SARS-CoV-2 lineage B.1.1.7. Nature. 2021;593:270–4. 33723411 10.1038/s41586-021-03426-1 PMC9170116

[28] Grint DJ , Wing K, Williamson E et al. Case fatality risk of the SARS-CoV-2 variant of concern B.1.1.7 in England, 16 November to 5 February. Euro Surveill. 2021;26(11):pii=2100256. 10.2807/1560-7917.ES.2021.26.11.2100256 PMC7976383 33739254

[29] Challen R , Brooks-Pollock E, Read JM et al. Risk of mortality in patients infected with SARS-CoV-2 variant of concern 202012/1: matched cohort study. BMJ. 2021;372:n579. 33687922 10.1136/bmj.n579 PMC7941603

[30] Abu-Raddad LJ , Chemaitelly H, Butt AA. Effectiveness of the BNT162b2 Covid-19 vaccine against the B. 1.1. 7 and B. 1.351 variants. N Engl J Med. 2021;385:187–9. 33951357 10.1056/NEJMc2104974 PMC8117967

[31] Lopez Bernal J , Andrews N, Gower C et al. Effectiveness of Covid-19 vaccines against the B. 1.617. 2 (delta) variant. N Engl J Med. 2021;385(7):585–94. 34289274 10.1056/NEJMoa2108891 PMC8314739

[32] World Bank . Data. https://data.worldbank.org/indicator/ [accessed December 21, 2021].

[33] World Health Organization . Maternal, newborn, child and adolescent health and ageing. Data portal. https://platform.who.int/data/maternal-newborn-child-adolescent-ageing/indicator-explorer-new/ [accessed December 21, 2021].

[34] Organization for Economic Cooperation and Development . OECD Stat. https://stats.oecd.org/Index.aspx [accessed December 21, 2021].

[35] Hale T , Angrist N, Goldszmidt R et al. A global panel database of pandemic policies (Oxford COVID-19 Government Response Tracker). Nat Hum Behav. 2021;5:529–38. 33686204 10.1038/s41562-021-01079-8

[36] Wadman M . Israel's grim warning: delta can overwhelm shots. Science. 2021;373(6557):838–9. 34413215 10.1126/science.373.6557.838

